# Effects of filtering by Present call on analysis of microarray experiments

**DOI:** 10.1186/1471-2105-7-49

**Published:** 2006-01-31

**Authors:** Jeanette N McClintick, Howard J Edenberg

**Affiliations:** 1 Department of Medical and Molecular Genetics, Indiana University, Indianapolis, Indiana, USA; 2 Center for Medical Genomics, Indiana University, Indianapolis, Indiana, USA; 3 Department of Biochemistry and Molecular Biology, Indiana University, Indianapolis, Indiana, USA

## Abstract

**Background:**

Affymetrix GeneChips^® ^are widely used for expression profiling of tens of thousands of genes. The large number of comparisons can lead to false positives. Various methods have been used to reduce false positives, but they have rarely been compared or quantitatively evaluated. Here we describe and evaluate a simple method that uses the detection (Present/Absent) call generated by the Affymetrix microarray suite version 5 software (MAS5) to remove data that is not reliably detected before further analysis, and compare this with filtering by expression level. We explore the effects of various thresholds for removing data in experiments of different size (from 3 to 10 arrays per treatment), as well as their relative power to detect significant differences in expression.

**Results:**

Our approach sets a threshold for the fraction of arrays called Present in at least one treatment group. This method removes a large percentage of probe sets called Absent before carrying out the comparisons, while retaining most of the probe sets called Present. It preferentially retains the more significant probe sets (p ≤ 0.001) and those probe sets that are turned on or off, and improves the false discovery rate. Permutations to estimate false positives indicate that probe sets removed by the filter contribute a disproportionate number of false positives. Filtering by fraction Present is effective when applied to data generated either by the MAS5 algorithm or by other probe-level algorithms, for example RMA (robust multichip average). Experiment size greatly affects the ability to reproducibly detect significant differences, and also impacts the effect of filtering; smaller experiments (3–5 samples per treatment group) benefit from more restrictive filtering (≥50% Present).

**Conclusion:**

Use of a threshold fraction of Present detection calls (derived by MAS5) provided a simple method that effectively eliminated from analysis probe sets that are unlikely to be reliable while preserving the most significant probe sets and those turned on or off; it thereby increased the ratio of true positives to false positives.

## Background

Affymetrix GeneChips^® ^are routinely used to measure relative amounts of mRNA transcripts on a genome wide basis. The large number of probe sets (representing genes) available on these arrays gives the researcher a wealth of information, but the multiple testing raises the potential for a large number of false positives. False positives and false negatives can both pose problems for the researcher, each with its own cost, so the balance between the two should be evaluated based upon the goals of the experiment. Increasing the stringency for accepting differences as significant (decreasing p-value) reduces false positives, which is important if verification and follow-up are costly, but simultaneously reduces true positives and may lead investigators to miss important trends in the data. Measurements of false positive risk, such as false discovery rate (FDR) [1,2], are now commonly used to help guide decisions. Although FDR gives the investigator an estimate of how many false positives to expect, it does nothing to identify which results are false positives.

Methods that differentially eliminate data that are likely to be unreliable can be of great help to the investigator. Not all genes are expected to be expressed at levels that are either biologically significant or detectable by the Affymetrix technology (1–3 copies per cell) in any particular tissue; in fact, the subset of genes expressed is what determines the characteristics of each tissue. For example, Jongeneel, *et al*. [3] estimated that 10,000–15,000 transcripts are expressed in human cell lines at one copy per cell or above. Data for genes not actually expressed represent experimental noise and cannot increase true positives, but can (and do) generate false positives. Discarding data for genes that are not expressed at detectable levels is, therefore, justified by biology and should result in an improvement in the balance between true and false positives.

Each Affymetrix GeneChip^® ^probe set contains 8 to 16 paired perfect match (PM) and mismatch (MM) 25-mer probes, which are used to determine whether a given gene is expressed and to measure the expression level (signal) [4]. The Affymetrix Microarray Suite version 5 (MAS5) algorithm uses the probe-pair data in different ways to calculate the detection call and the signal. MAS5 uses a non-parametric statistical test (Wilcoxon signed rank test) of whether significantly more perfect matches show more hybridization signal than their corresponding mismatches to produce the detection call (Absent (A), Present (P) or Marginal (M)) for each probe set [5]. We will use the convention of capitalized Present, Absent, and Marginal to indicate the formal detection calls. The signal is the anti-log of an average (Tukey biweight) of the log(PM-MMadj) for all of the probe pairs, where MMadj is equal to MM or an adjusted quantity which will produce a positive value [6]. Genes that are not detectably expressed nevertheless generate signal values, usually low; random fluctuations in these low values can often produce large apparent fold-changes.

Different methods have been used to pre-filter data to remove data for probe sets that are believed to be less reliable, but the effects of such pre-filtering are rarely analyzed quantitatively. Filtering by signal (expression level) [7] removes probe sets with signal close to background; the choice of how close to background is arbitrary. Removal of probe sets that are called Absent on all arrays has been reported [8]. Some use post-hoc methods by eliminating significant probe sets with low fold changes [9]; again, the choice of fold-change is arbitrary and there is no theoretical null distribution. Others use combinations of these strategies: trimming upper and lower signals plus fold change filter [10]; confidence score based on fold change, p-value, signal and percent Present [11]; signal, fold change and percent Present filters [12]; minimum signal and fold change [13]. Most of these studies did not provide a reference for their selection of filtering criteria or provide evidence that these strategies were helpful. Aston *et al*. [12] chose their fold-change threshold based on fold changes seen in previous microarray studies of post-mortem brain tissue, but technical variations between experiments can affect the distribution of signals. Stossi *et al*. [11] provided two references for their confidence score but neither of these gave a rationale for the calculation. Seo, *et al. *demonstrated that using the MAS5 detection p-value as a weighting factor in the distance measures for hierarchical clustering increased the ability to separate samples from biologically different groups [14].

We have filtered out probe sets that were not called Present by the MAS5 detection call in at least 50% of the samples in one treatment group [15,16]. Requiring the probe set to meet the criterion in any one treatment group retains genes that are turned on or off; these are usually very interesting to biologists. Our method, which we call filtering by fraction Present, improves FDR. Here, we evaluate the effects of setting different thresholds and compare this approach to the use of thresholds for signal values. These analyses were performed using expression level data generated by two widely used algorithms, MAS5 and RMA (robust multichip average [17,18]). RMA is an alternative log scale measurement of expression derived from only the perfect match probes by fitting them to a linear model normalized across all arrays in the experiment. RMA does not provide a measure comparable to the MAS5 detection call. We examined the effects of the fraction Present filtering on the ability to detect changes in expression using a parametric t-test and also using a permutation-based test, significance analysis of microarrays (SAM) [19]. We also explore how experiment size can affect the ability to detect differences in gene expression, and how the sample size interacts with the choice of filtering thresholds, using permutations of the full data set to create virtual experiments of smaller sample sizes. These analyses provide useful guidelines for improving the design and interpretation of microarray experiments.

## Results

### Distribution of signal and RMA values

**Figure 1:**
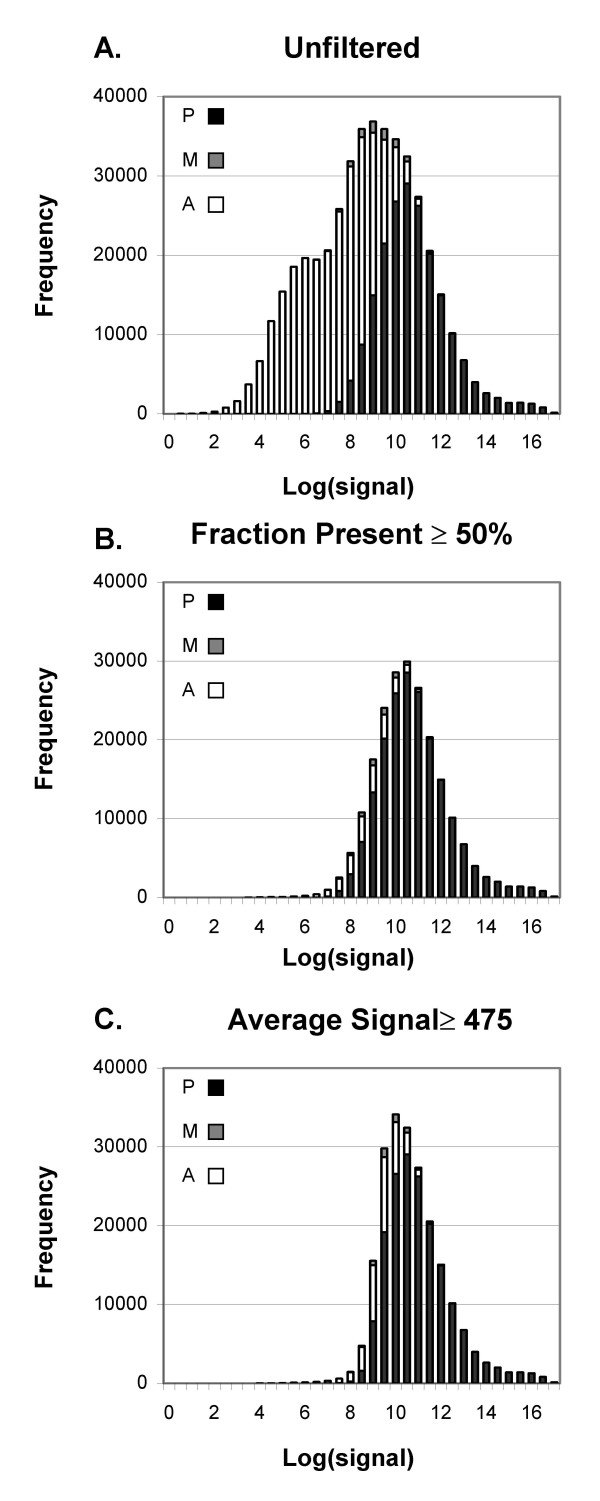
**Distribution of MAS5 log_2_(signals) before and after filtering**. A) No filter. B) Filtering with threshold of ≥ 50% Present in at least one treatment group. C) Filtering by average signal with threshold at ≥475 in at least one treatment group. The number of probe-sets at each value of Log2(signal) are plotted. Black = Present, gray = Marginal, white = Absent.

The *a priori *expectation is that not all genes are expressed in any given tissue at levels detectable by Affymetrix GeneChip^® ^arrays (1–3 transcripts per cell) [3]. It therefore makes both biological and statistical sense to avoid analyzing differences in the apparent expression of genes that are not truly expressed. To compare two methods of filtering out such probe sets, we examined a dataset that compares 10 arrays from cells without interferon to 10 arrays from cells treated with interferon alpha (IFN dataset [16]). Of the 445,660 individual probe sets (20 arrays with 22,283 probe sets per array) in the IFN data set, 54% were called Absent by the MAS5 algorithm. There is a strong suggestion of bimodality in the distribution of log_2_(signal) for all probe sets (Fig. 1A), with a large number of Absent probe sets forming a shoulder at low signal values. Removing probe sets in which fewer than half of the arrays in at least one of the experimental groups was called Present eliminates the large shoulder of Absent probe sets, leaving a distribution that is more nearly normal (Fig. 1B). It is important to note that the signals for all samples in a particular probe set are treated as a group; all are retained or all removed based on whether at least one experimental group (interferon treated or control, in this example) meets the filtering criterion. In Fig. 1, signal values for probe sets for *individual *samples are shown, hence the retention of some Absent samples after filtering. An alternative method of removing probe sets thought to represent genes with little or no expression is to set a minimum average signal level for at least one of the two experimental groups. Fig. 1C shows the distribution of signal values when a minimum average signal of 475 is required; 475 was chosen to retain a similar number of probe sets as the 50% Present filter shown in Fig. 1B. Filtering based on the signal values more severely truncated the low expression values (Fig. 1C).

**Figure 2:**
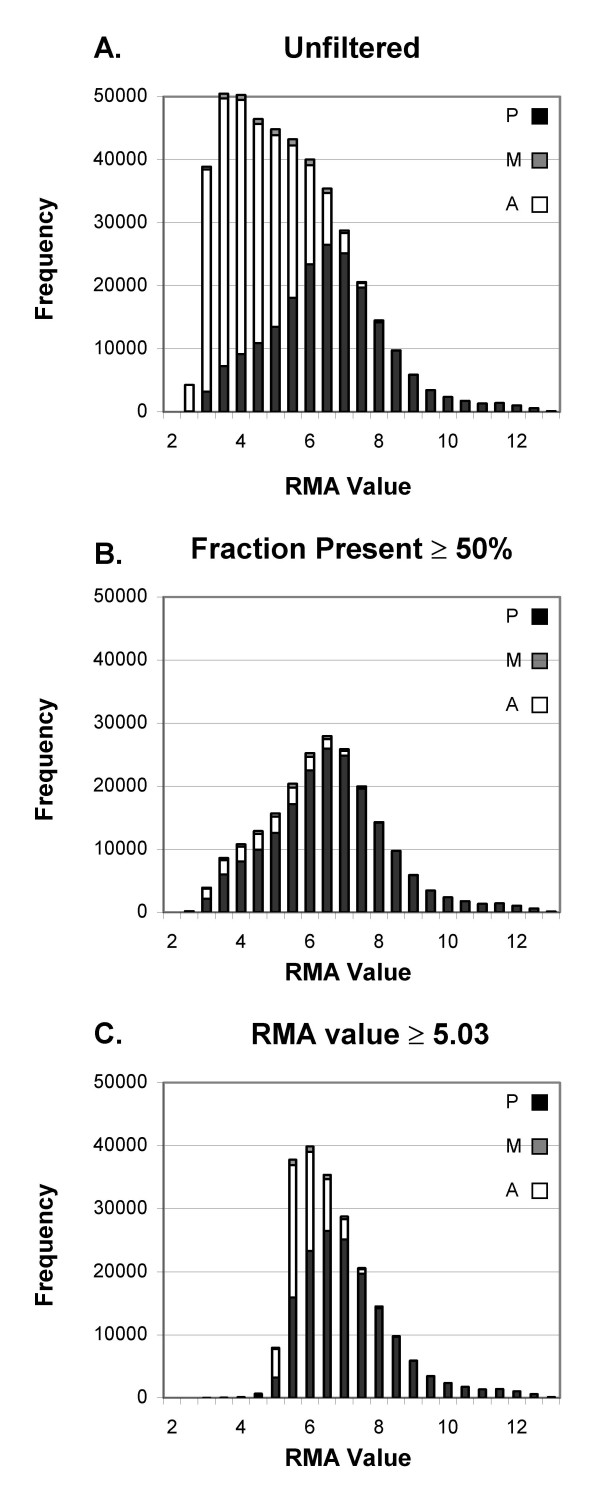
**Distribution of RMA values before and after filtering**. A) No filter. B) Filtering with threshold of ≥ 50% Present in at least one treatment group. C) Filtering by average RMA value with threshold at ≥5.03 in at least one treatment group. Symbols as in Fig. 1.

The distribution of RMA values (Fig. 2A) has a greater abundance of probe sets with low values than does the MAS5 signal, and more of the probe sets called Present have low values (compare Figs. 1A and 2A). Filtering by a threshold of 50% Present leaves many of the low-level Present probe sets (Fig. 2B). In contrast, filtering by the average RMA value ≤5.03, which removes a comparable number of probe sets, removes the low probe sets and truncates the left-most portion of the graph (Fig. 2C).

### Effects of different filtering thresholds

**Figure 3:**
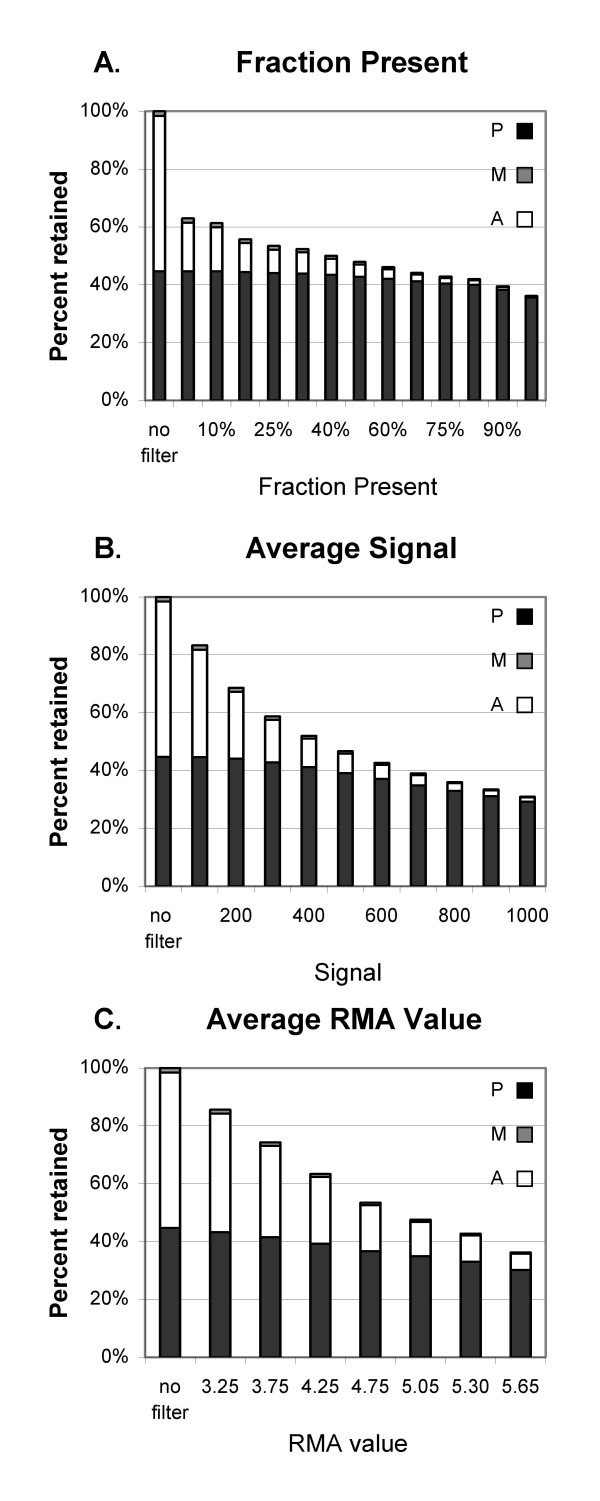
**Percent of probe sets remaining after filtering**. Percent of probe sets remaining after filtering using selected thresholds for A) Fraction Present. B) MAS5 Signal. C) RMA value.

We explored the effect of setting different thresholds for both types of filtering on the percentage of probe sets retained that have each detection call (A, P, M). When adjusted to remove comparable numbers of probe sets, filtering by fraction Present does better at retaining probe sets called Present. About 37% of the probe sets are Absent on all 20 arrays, and are therefore removed by the fraction Present filter set to any fraction greater than 0 (Fig. 3A). No Present probe sets were removed until the threshold was at least 20%, by which point 81% of the Absent probe sets were removed. Setting the fraction Present to 50% removes 4.2% of the Present probe sets along with 92% of the Absent probe sets (Fig. 1B and 3A). Filtering by either signal value (Fig. 3B) or RMA value (Fig. 3C), when adjusted to remove a comparable number of probe sets, leaves a less favorable balance between Absent and Present probe sets. Unlike filtering by fraction Present, signal-based filtering removes Present probe sets even when using a very low minimum MAS5 signal: a minimum MAS5 signal of 254 leaves the same number of probe sets as the Present filter set at >0%; at that signal threshold, 2.7% of the Present probe sets are removed along with 66% of the Absent probe sets. For RMA the balance between removing Absent and Present probe sets is even worse: the equivalent threshold of 4.25 removes 12.1% of Present probe sets and 57% of Absent. As the signal value is increased beyond that, a greater fraction of Present probe sets is removed. Average Signal or RMA value thresholds that leave a number of probe sets equal to the results of using 50% Present remove 11.3% and 21.6% of Present probe sets while only removing 86% and 77% of the Absent probe sets (for MAS5 signal or RMA value, respectively; Figs. 3B, 3C).

**Figure 4:**
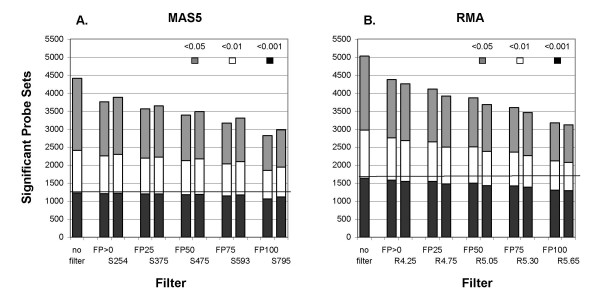
**Number of significant probe sets after filtering**. A) Filtering by fraction Present *vs. *by average MAS5 signal. The probe sets called significantly different (at the p-values shown) between the interferon treated and untreated samples in the 10 sample experiment are plotted against the threshold of Fraction Present (FP) or average signal (S), followed by threshold value. The horizontal line at 1230 indicates the number of probe sets at p ≤ 0.001 in the unfiltered data. Paired thresholds remove comparable numbers of probe sets, e.g. FP>0 and S254. B) Filtering by fraction Present *vs*. by average RMA value. (FP) Fraction Present, (R) average RMA value, followed by threshold value. The line at 1641 indicates the number of probe sets at p ≤ 0.001 in the unfiltered data.

There was substantial agreement among the filtering methods in which probe sets were retained when the thresholds were chosen to retain comparable numbers of probe sets (Table 1). Between 85 and 89% of the probe sets retained with the fraction Present filter were also retained when filtering by minimum MAS5 signal. The agreement was less when filtering by RMA values, with only 77% of the probe sets in common. Similar findings are seen for the vitamin A data (Table 1). Very few probe sets with p ≤ 0.001 are lost by filtering and the overlap between signal and fraction Present filtering is better with MAS5 data than RMA data (Table 2). [Note that the signal and RMA values that remove similar numbers of probe sets are quite different for the vitamin A data than for the IFN data.] Increasing the stringency of filtering decreases the number of probe sets with nominally significant p-values, as expected, but the more significant probe sets (p ≤ 0.01 and p ≤ 0.001) are the least affected (Fig. 4, Table 2).

**Table 1:** Probe sets remaining at different filtering thresholds^a^.

	**Unfiltered (total)**	**FP>0** **sig254** **RMA4.25**	**FP25** **sig375** **RMA4.75**	**FP50** **sig475** **RMA5.03**	**FP75** **sig593** ** RMA5.30**	**FP100** **sig795** **RMA5.65**
**IFN Data**						
Fraction Present	22,283	14,035	11,906	10,678	9,545	8,050
Average Signal	22,283	14,030	11,888	10,657	9,546	8,057
Overlap (MAS5)^b^		89%	89%	88%	87%	85%
Average RMA value	22,283	14,118	11,909	10,687	9,526	8,076
Overlap (RMA)^c^		77%	77%	77%	77%	77%

	**Unfiltered (total)**	**FP>0** **sig200** **RMA5.05**	**FP25** **sig265** **RMA5.47**	**FP50** **sig323** **RMA5.70**	**FP75** **sig400** ** RMA5.95**	**FP100** **sig475** **RMA6.17**

**Vitamin A Data**						
Fraction Present	8799	4611	3981	3627	3237	2896
Average Signal	8799	4536	3988	3625	3231	2904
Overlap (MAS5)^b^		84%	84%	84%	83%	93%
Average RMA value	8799	4639	3984	3635	3229	2890
Overlap (RMA)^c^		73%	72%	72%	72%	71%

^a^For comparison of different filters, thresholds were selected for each filter that removed similar number of probe sets. Types of filter: Fraction Present, 'FP' followed by threshold value; Average MAS5 signal, 'sig' followed by signal threshold; average RMA value, 'RMA' followed by threshold value. ^b^The percent of probe sets in common retained by both fraction Present and average MAS5 signal filter. ^c^The percent of probe sets in common retained by both fraction Present and average RMA value filter.

**Table 2:** Highly significant (p ≤ 0.001) probe sets lost at different filtering thresholds^a^.

	**Unfiltered (total)**	**FP>0** **sig254** **RMA4.25**	**FP25** **sig375** **RMA4.75**	**FP50** **sig475** **RMA5.03**	**FP75** **sig593** ** RMA5.30**	**FP100** **sig795** **RMA5.65**
**IFN Data**						
Fraction Present (MAS5)	1230	20	30	49	84	165
Average Signal (MAS5)	1230	8	26	43	57	112
Fraction Present (RMA)	1641	53	92	143	218	334
Average RMA (RMA)	1641	92	161	205	252	346

	**Unfiltered (total)**	**FP>0** **sig200** **RMA5.05**	**FP25** **sig265** **RMA5.47**	**FP50** **sig323** **RMA5.70**	**FP75** **sig400** ** RMA5.95**	**FP100** **sig475** **RMA6.17**

**Vitamin A Data**						
Fraction Present (MAS5)	168	8	10	10	14	17
Average Signal (MAS5)	168	5	9	13	19	24
Fraction Present (RMA)	177	5	7	10	14	18
Average Signal (RMA)	177	13	17	22	28	31

^a^For comparison of different filters, thresholds were selected for each filter that removed similar number of probe sets. Types of filter: Fraction Present, 'FP' followed by threshold value; Average MAS5 signal, 'sig' followed by signal threshold; average RMA value, 'RMA' followed by threshold value. Total number of probe sets with p ≤ 0.001, followed by the number removed at each threshold (cumulative).

**Figure 5:**
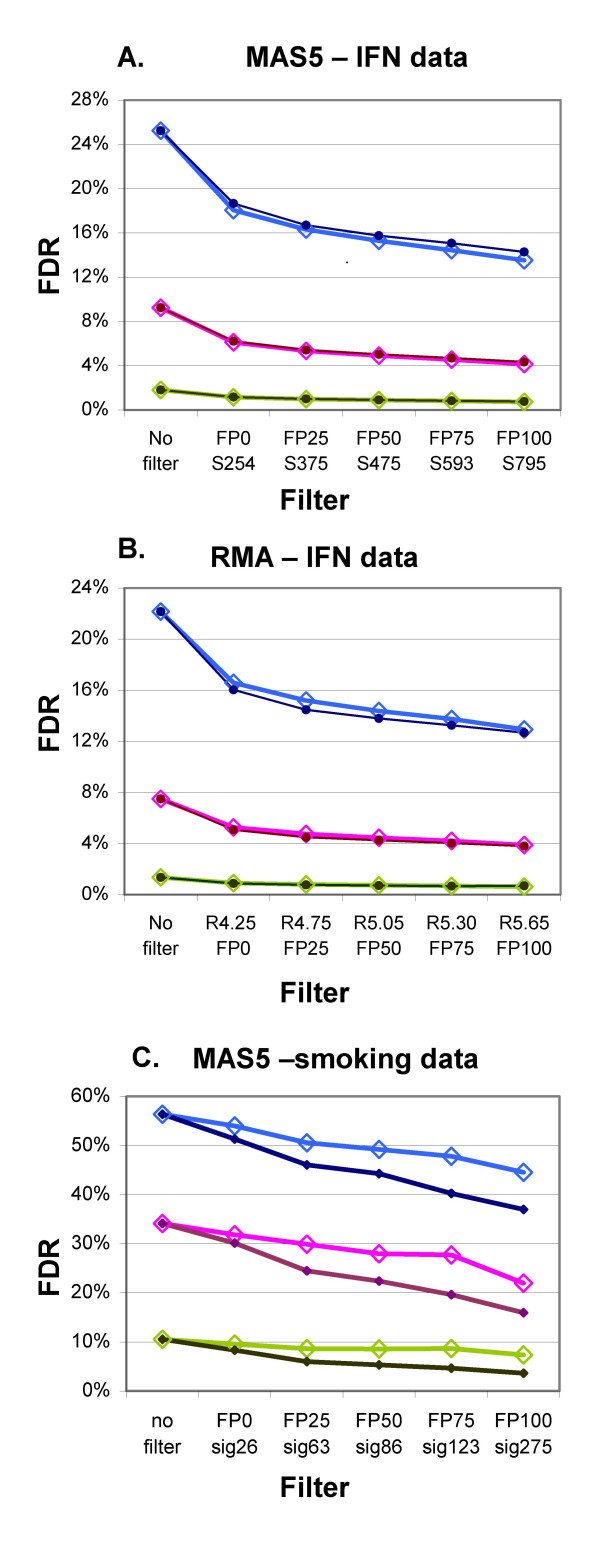
**Effect of Filtering on false discovery rate (FDR)**. Filter method and values (x-axis): Fraction Present (FP), signal (S) or RMA value (R) followed by threshold value; separate lines are shown for each. Closed circles represent values from fraction Present filtering, open diamonds from average signal or average RMA. P-values: 0.05 (blue), 0.01 (pink), and 0.001 (green). A) IFN data, MAS5, B) IFN data, RMA, C) Smoking data, MAS5. Note that the smoking data was scaled to 100 instead of 1000 used for the other data sets.

The FDR for any particular p-value is an estimate of the percentage of probe sets expected to be false positives for the group of probe sets that have a p-value less than or equal to the selected p-value. FDR is based on the number of probe sets used in the analysis (retained after the filtering). Filtering by fraction Present or by signal or RMA value have similar effects on the FDR calculated according to Benjamini and Hochberg [1] (Fig. 5). The largest stepwise improvement in FDR occurs with the initial filtering, Present fraction > 0% or removal of an equivalent number of probe sets by an average MAS5 signal ≥ 254 or RMA value ≥ 4.25. Increasing the stringency of the filter continues to improve the FDR. However, in the IFN experiment (10 arrays in each group), increasing stringency beyond 25% Present, MAS5 signal ≥ 375, or RMA value ≥ 4.75 leads to little improvement in FDR for the more significant probe sets (p ≤ 0.001), while increasing the number of those very significant probe sets lost (Table 2). Results for the smaller vitamin A data seta are similar (Table 2). The smoking dataset (20 arrays per group) has higher variability within each group. In those data, the fraction Present filter shows a larger advantage over the average signal filter, which increases with increasing stringency (Fig. 5C).

The Benjamini and Hochberg [1] FDR is conservative. An alternative FDR algorithm described by Storey and Tibshirani [2] produced FDR estimates that were about 50% better (smaller) than those shown in Fig. 5A. Like the Benjamini and Hochberg FDR, the Storey FDR was improved by more than 50% when the data were filtered using a threshold of 25% Present.

### Permutation analyses

We used permutations of the IFN data in which 5 samples from each treatment group were combined to produce two new groups expected to show no difference. Welch's t-tests on the MAS5 log transformed data produced fewer nominally significant probe sets than expected by chance (Table 3): 0.04 at a nominal p = 0.05 and 0.0005 at a nominal p = 0.001. In these balanced permutations, 37% of probe sets are Absent on all arrays but 43% of the probe sets with p ≤ 0.05 are found in this "all-Absent" group, demonstrating that the Absent probe sets make a disproportionate contribution to false positives. Similar analyses of the smoking data (Fig. 5C) also produced fewer nominally significant probe sets than expected by chance.

**Table 3:** Average fraction of false positives: permutation tests.

	**Nominal P-value**		
	**0.050**	**0.010**	**0.0010**

**Unfiltered**	0.039	0.007	0.0005
**All Absent**	0.045	0.008	0.0007
**Fraction Present >0**	0.035	0.006	0.0005

Permutation of the MAS5 data such that no true positives would be expected were carried out 1000 times. Results shown are the average fraction called significant by the Welch's t-test for selected p-values (0.05, 0.01 and 0.001). Results are for unfiltered data, probe sets called Absent for all samples (37% of the probe sets), and data filtered using fraction Present >0.

To determine if filtering would also improve other types of analyses, we analyzed the IFN MAS5 data either unfiltered or after filtering by fraction Present with thresholds of >0%, 25% and 50% using Significance Analysis of Microarrays (SAM) [19]; SAM uses permutations to calculate an FDR (q-value). Filtering by fraction Present increased the number of probe sets deemed significant at each FDR, demonstrating that filtering is beneficial for this alternate method of determining significant changes. Comparing fraction Present >0% to unfiltered data, there was a 35% improvement in the number of probe sets significant at 1% FDR. In this 10-sample experiment, more restrictive filtering (25% and 50%) did not result in appreciable improvement over >0% Present.

### Effects on genes turned on or off

The most pronounced difference between filtering by fraction Present and by average signal is the effect on probe sets that are turned "on" or "off". Genes that are turned on or off are of particular biological interest. In the IFN data there are 108 probe sets that met our criteria for being turned on or off (Table 4); there are 31 in the vitamin A data. Filtering by fraction Present, none of these probe sets were removed when the threshold was ≤ 50% for either data set. Filtering by signal value, on the other hand, removed "on/off" probe sets at each of the selected thresholds (Table 4). Demanding that all probe sets be Present in one of the two conditions leads to a 70% decrease in the detection of probe sets turned on or off in IFN data and an approximately 50% decrease for the vitamin A data.

**Table 4:** Number of on/off probe sets retained after filtering at selected thresholds.

**IFN data**	**Unfiltered**	**FP>0** **sig 254**	**FP25** **sig375**	**FP50** **sig475**	**FP75** ** sig593**	**FP100** **sig795**
Fraction Present	108	108	108	108	90	34
Average Signal	108	104	100	89	81	64

**Vitamin A data**		**FP>0** **sig 200**	**FP25** **sig265**	**FP50** **sig323**	**FP75** **sig400**	**FP100** **sig475**

Fraction Present	31	31	31	31	27	16
Average Signal	31	26	22	17	16	13

Filtering by Fraction Present (FP) and average MAS5 signal (sig) followed by threshold. On/off: probe sets with p < 0.05 for t-test and in which the sum of Present calls differed by more than 6 for IFN data and more than 4 for vitamin A data between the two treatment groups were called turned "on" or "off".

### Effects of using fraction present in entire experiment rather than by treatment group

Our approach of requiring at least one group to meet the threshold insures that the gene is expressed at a detectable level in at least one biologically defined group; that seems *a priori *preferable to requiring a (lower) fraction present across the entire experiment, because the latter could include genes that are not reliably detected in any biological condition. To examine whether our choice led to a significant bias, we compared these two approaches. Using a fraction Present >0 (requiring just a single Present array) is, of course, identical for both methods. Comparing thresholds of 25% or 50% Present in at least one group (our method) with thresholds across the entire experiment that led to retention of similar numbers of probe sets, the number significant at any particular p-value was within 2%. filtering based on fraction Present in at least one group was much better at preserving probe sets turned on or off than using a global threshold.

### Effects of sample size on power

**Figure 6:**
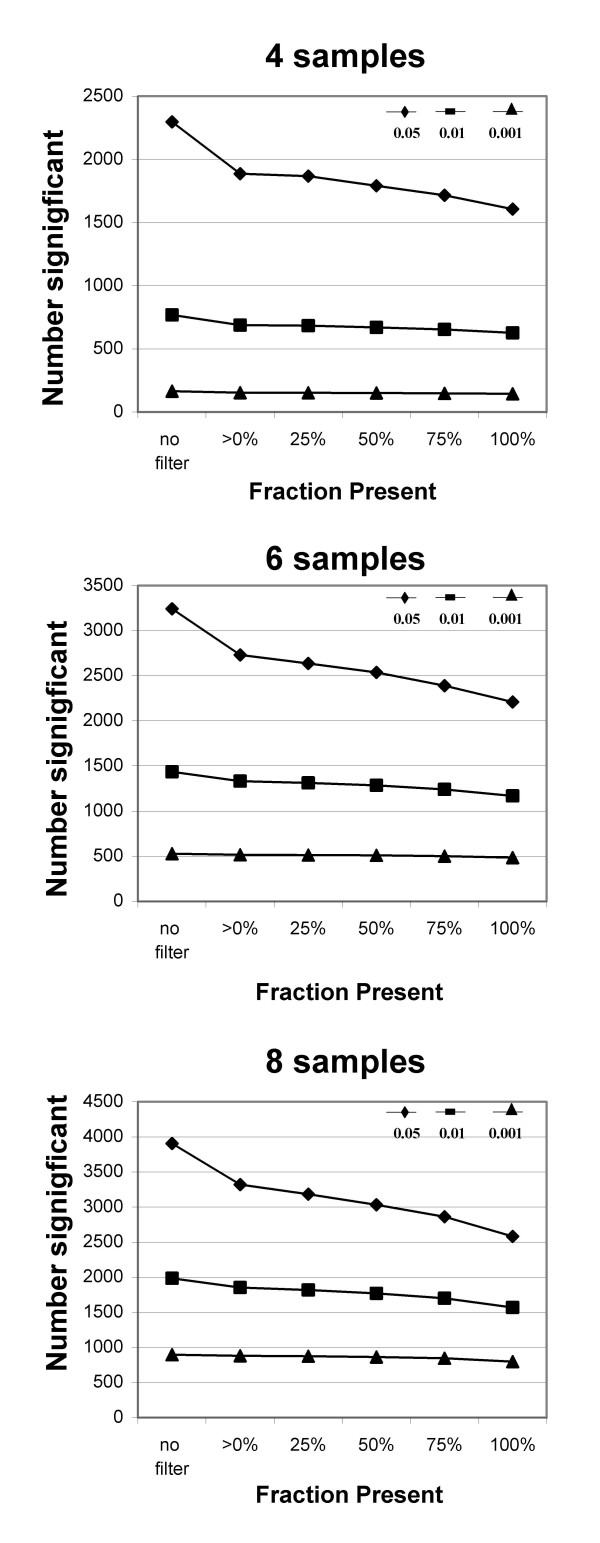
**Effect of filtering on average number of significant probe sets in smaller experiments**. Smaller virtual experiments (4, 6 and 8 samples per treatment group) were created by random selection of arrays within each of the two treatment groups (based on 1000 permutations). The probe sets called significantly different (at the p-values shown) are shown for different values of fraction Present (x-axis). Note differences in scale for y-axes of the 3 graphs. P-values: ≤ 0.05 diamond, ≤0.01 square, ≤0.001 triangle.

**Figure 7:**
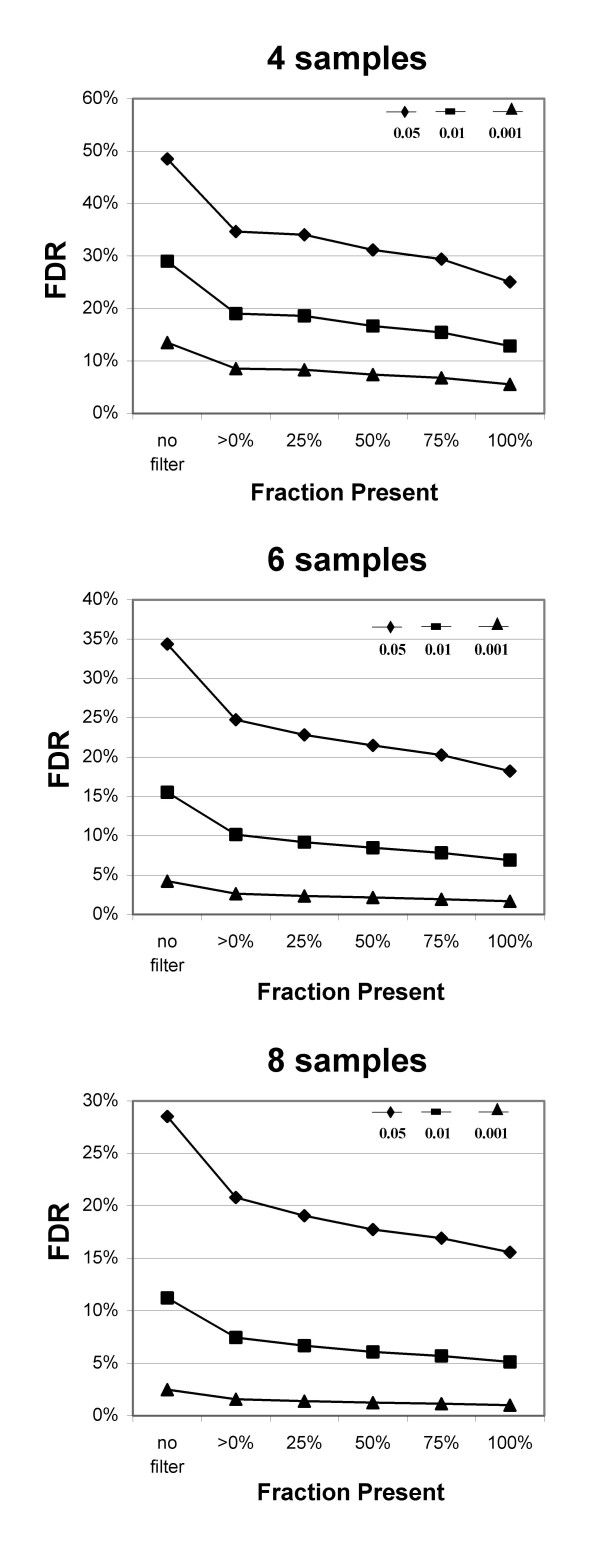
**Effects of filtering on FDR in smaller experiments**. FDR for the smaller virtual experiments shown in Fig. 6. Note differences in scale for y-axes of the 3 graphs. P-values: ≤ 0.05 diamond, ≤0.01 square, ≤0.001 triangle.

Most of the experiments completed by our core facility have fewer than 10 samples per treatment group, typically 4–6, and some in the literature use fewer than 4. To understand the effects of sample size on filtering we created smaller virtual experiments (with 3, 4, 5, 6, 7, and 8 samples per treatment group) from the IFN data by using permutations of the original data. We randomly selected arrays without replacement within each treatment group; this created virtual experiments in which a difference in expression is expected. There is a large increase in power as the sample size increases (Fig. 6) especially for the most significant probe sets (p ≤ 0.001): a 15-fold increase from 3-sample experiments to 8-sample experiments. The effects of filtering in the smaller experiments were similar to the effects seen in the full experiment (Fig. 6). The more significant probe sets (p ≤ 0.001) were mostly retained when filtering was at ≤ 50% Present (Table 5). The FDR is similarly improved by filtering (Fig. 7; note the differences in scales in each panel for Figs. 6 and 7). Similar permutations were performed using the Smoking data for samples sizes of 6, 10 and 14. The number of probe sets with p ≤ 0.001 increased nearly 3-fold going from 6 to 10 samples; increasing to 14 sample added another 70% and the full 20 samples had a further 60% improvement over 14.

**Table 5:** Number of very significant probe sets lost after filtering in smaller experiments.

	**Probe sets at p ≤ 0.001**	**Probe sets at p ≤ 0.001 lost by filtering at selected thresholds**				
**# samples**	**Unfiltered**	**> 0%**	**≥ 25%**	**≥ 50%**	**≥ 75%**	**100%**
**3**	59	10	10	11	12	13
**4**	165	12	12	14	16	20
**5**	333	12	14	16	21	28
**6**	528	12	14	18	27	43
**7**	718	14	17	25	40	68
**8**	897	16	21	32	50	99

Number of probe sets at p ≤ 0.001 found in the unfiltered IFN data, and the number of those lost by filtering at each of several fraction Present thresholds, for experiments with 3 to 8 samples per treatment group.

We carried out a second set of permutations this time randomly selecting equal numbers of samples from each treatment group to create two new groups expected to produce no true positives. For these tests of 4, 6 and 8 samples in each treatment group, a disproportionate number of the false positives were found in the probe sets that are Absent for all arrays (Table 6). the number found significant was less than expected for a normal distribution, results that are similar to the results of permutation tests on the full data set.

**Table 6:** Fraction of false positives in smaller experiments.

	**Unfiltered**			**All Absent**			**Fraction Present >0**		
**# samples**	**0.050**	**0.010**	**0.0010**	**0.050**	**0.010**	**0.0010**	**0.050**	**0.010**	**0.0010**
**4**	0.029	0.005	0.0005	0.035	0.007	0.0009	0.030	0.005	0.0004
**6**	0.036	0.006	0.0005	0.041	0.007	0.0007	0.033	0.005	0.0004
**8**	0.039	0.006	0.0050	0.044	0.008	0.0007	0.035	0.006	0.0004

Permutations generating smaller experiments with 4, 6, or 8 samples per treatment group of the IFN MAS5 data such that no true positives would be expected were carried out 1000 times. Results shown are the average fraction called significant by the Welch's t-test for selected p-values (0.05, 0.01 and 0.001). Results are given for unfiltered data, probe sets called Absent for all samples, and data filtered using fraction Present >0.

### Effect of sample size and filtering on consistently significant probe sets

**Figure 8:**
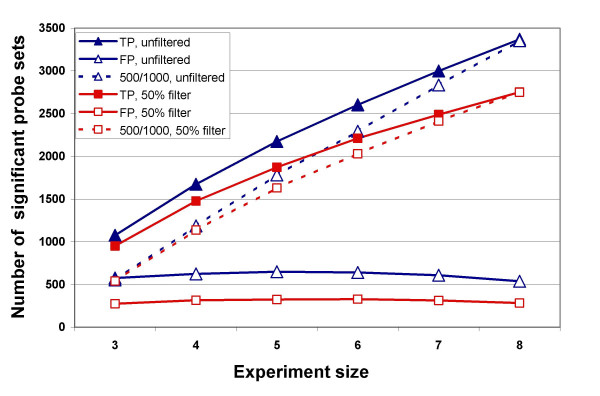
**Effect of experiment size on true positives, false positives and consistent positives**. TP: true positive, p-value ≤ 0.05 in smaller simulated experiment and p ≤ 0.05 in full 10-sample analysis. FP: false positive, p-value ≤ 0.05 in smaller simulated experiment but p > 0.05 in full 10-sample analysis. 500/1000: consistent positives, found significant at p < -0.05 in at least 50% of the 1000 permutations. Data are shown both unfiltered and after filtering by 50% Present.

While there is no gold standard for the IFN data, the full 10-sample dataset provides a reasonable standard to which the smaller virtual experiments can be compared. The Benjamini and Hochberg [1] FDR for the full experiment (without filtering) at p = 0.05 is 25%, the Storey [2] FDR is 17%, and the estimate by permutation is 20%. Therefore, a crude estimate of false discovery can be made by comparing the results from the small experiments to the results using all samples, assuming that any probe set that was called significant in the smaller experiments but had a p-value > 0.05 in the full experiment (without filtering) was a false positive. The number of false positives identified in this manner is slightly smaller than was found by permutations, and remains about constant for experiments of size 3 to 8 samples per treatment group (Fig. 8). The number identified as "true positives" (i.e. matching the full 10 sample experiment) at p ≤ 0.05 increases with the size of the experiment. Filtering at 50% Present removes about 50% of the false positive probe sets across the full range of sizes, and removes from 11.6% (3 samples) to 18.4% (8 samples) of the true positive probe sets (primarily those with p > 0.01). This suggests that larger experiments do not need as stringent a fraction Present filter.

Another way of analyzing the likely true positives in smaller experiments is by examining the reproducibility of results. We assumed that probe sets deemed significant at least 50% of the time (*i.e. *in ≥ 500/1000 permutations) represents consistent, reproducible data. The number of consistently significant probe sets increases as the experiment size increases and approaches the number identified as true positive probe sets (Fig. 8). In the smaller virtual experiments (3 or 4 samples per treatment group) only 2–4% of the probe sets consistently significant at p ≤ 0.05 are lost when filtering by 50% Present. In larger experiments (5 or 6 samples per group) 6–9% of these consistently significant probe sets were lost when filtering by 25% Present (Fig. 8). For those probe sets consistent at p ≤ 0.01, the number lost by filtering is even smaller, almost none for 3–4 samples at 50% Present and 1–2% for 5–6 samples per group at 25% Present. Only 2 probe sets in the 3-sample permutations were found to be significant at p ≤ 0.001 at least 50% of time, whereas 65 such probe sets were found in the 4-sample permutations. All of these were retained by fraction Present filtering at all thresholds.

In contrast to removing probe sets that represent genes not expressed, setting a fold change limit by itself does not appear to increase the likelihood that a change is in fact real. Absent probe sets with low signals have an increased probability of generating spurious large fold changes, especially in smaller experiments (3–5 samples). For these small experiments, the probe sets remaining after 50% Present filter generate a modest number of fold changes ≥2 of which 71–84% are called significant in the full 10-sample experiment at p ≤ 0.01. On the other hand those probe sets removed by the 50% filter generate about 4 times as many fold changes ≥2, of which only 6 to 10% are called significant in the 10-sample experiment at p ≤ 0.01.

## Discussion

Microarray experiments allow one to examine global patterns of gene expression, but by their nature involve multiple comparisons that can generate false positives. While the idea of removing probe sets that are unlikely to produce positive results is not new, we present a systematic analysis of the effects of several strategies. Not all genes are expressed in any one tissue [3]. Probe sets that have very low signals or are called Absent primarily reflect noise in the data, and give a large number of false positives without adding many true positives. Permutations expected to produce no significant changes confirmed that Absent probe sets have an increased risk of producing false positives (Tables 3 and 6). Requiring that only one treatment group meet the threshold, particularly with our recommended filtering by Fraction Present, preserves data for genes that are turned on or off, genes that may be of great interest to biologists.

Filtering by fraction Present does a better job of removing most of the Absent probe sets while retaining most of the Present probe sets than filtering by either average MAS5 signal or RMA value (Figs. 1, 2 and 3), and results in much better FDR (Fig. 5, 7). Our main evaluation criteria, improvement of FDR, was chosen because this experiment-wide measure of confidence is widely applied. Because there is no "gold standard" for real experiments, we used measures that increased the likelihood of a result being a true positive, such as p-value < 0.001 for a Welch's t-test and consistency of detecting the difference in multiple permutations. Fraction Present filtering removes very few probe sets with p ≤ 0.001 (Fig. 4 and 6, Tables 2 and 5); it does not remove probe sets that are turned on or off unless the threshold is set above 50% Present (Table 4). Unlike using signal or RMA values, thresholds chosen for fraction Present are not affected by chip type, percent called present, method of scaling or normalization, nor by the method used to produce the expression value (e.g. MAS5, RMA).

Permutation of the IFN data to simulate smaller experiments also showed that the Absent probe sets generated a disproportionate number of false positives (Fig. 8, Table 6) many of which had fold changes larger than 2, showing that fold-change alone as a filter cannot fix this problem.

**Figure 9:**
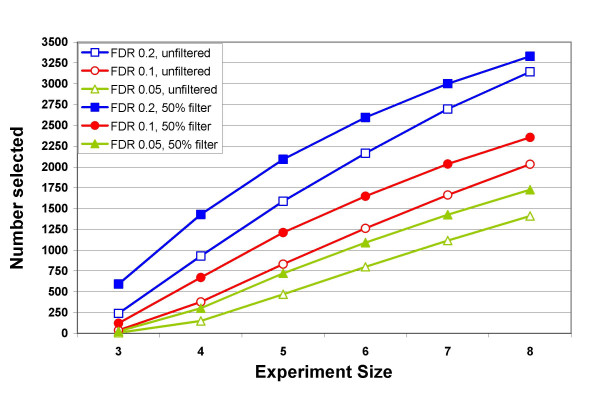
**Effect of experimental size on number of probe sets meeting a fixed value of FDR before and after filtering**. The number of probe sets meeting various Benjamini and Hochberg FDR thresholds, 0.2 (blue), 0.1 (red), and 0.05 (green) before (open symbols) and after filtering (filled symbols) by 50% Present. Number selected is average over 1000 permutations.

Filtering increased the average number of probe sets that met an FDR of 0.1 in the IFN data for experiments of all sizes (Fig. 9), and was particularly helpful for the smaller experiments: over 3-fold improvement for the 3 sample experiments (38 to 122) and nearly double for the 4 sample experiments (378 to 672). Small experiments (3–4 samples) have limited power to detect changes, and very few probe sets can be consistently identified (Fig. 8). Filtering by fraction Present greatly improves FDR even for small experiments, and retains nearly all of these reproducible probe sets (Figs. 7, 8). While we think that experiments should use more than 3 or 4 samples, this filtering method should improve results from small experiments such as pilot projects.

Pavlidis, *et al. *[20] demonstrated that 10 to 15 samples (or fewer in some cases) produced reproducible results, as determined by their stability measures of order and recovery. Our study demonstrates that even large experiments benefited from filtering by fraction Present; The IFN data with 10 samples and the smoking data with 20, both had improvements in FDR after filtering (Fig. 5), with approximately a 50% improvement in FDR when using a fraction Present of 0.25 for filtering.

Removing only those probe sets called Absent in all samples provides the single largest improvement in FDR and appears to be sufficient for large experiments. Although the FDR is somewhat better with more stringent filtering (Fig. 7), the loss of probe sets at p ≤ 0.001 indicates there may be an accelerated loss of true positives in the larger data sets (Table 5). as the experiment size decreases, the criterion for filtering should be increased (Fig. 6, 7; Table 5). For data sets with 3–4 samples 50% Present spares most of the probe sets significant at p ≤ 0.001 and those probes sets found most consistently (Fig. 6, 8). For more samples, relaxing the threshold to 25% fraction Present is reasonable (Fig. 6, 7, and 8). Requiring 100% Present in one of the two treatment groups is not recommended, because it removes too many highly significant probe sets (Tables 2 and 5) and removes a large portion of the probe sets turned on or off (Table 4) in experiments of any size.

The results are similar for datasets that differ greatly. The IFN data, presented in the most detail, was from an experiment that examined the effects of interferon treatment on human PBMC *in vitro *[16], and used the HGU133A GeneChip^®^. The vitamin A data compared RNA extracted from liver tissue from Sprague-Dawley rats fed vitamin A deficient or sufficient diets [15,21], and used the relatively old RGU34A GeneChip^®^, designed with much less sequence data and informatics. The smoking data are from a large study examining differences in bronchial epithelia extracted from human subjects [22], and also used HGU133A GeneChip^®^; the variability within each group in the smoking dataset is much greater than in the others. The Pearson correlation between samples from subjects within each of the two groups in the smoking data were 0.87 and 0.89, compared to an average Pearson correlation >0.97 for samples within each group for the IFN data. In all three cases, representing different generations of GeneChip^®^, different species, different laboratories and different amounts of intra-group variability, our approach achieved the primary goal of improving FDR while minimizing the removal of very significant probe sets (p ≤ 0.001) and retaining those probe sets turned on or off.

Filtering based on the fraction of Present calls is superior to methods based on signal or RMA value because it is more likely to preserve probe sets turned on or off and it removes probe sets that show cross-hybridization. Filtering by fraction Present is also much easier to implement, because general guidelines can be set based upon the experiment size instead of having to examine the distribution of signal values; the variability of the signal distributions for different datasets is such that no average signal value gives comparable results across all datasets (Table 1). Although the detection call is generated by MAS5, this method can be used as a pre-filter to improve results using non-MAS5 generated data, such as RMA.

## Conclusion

Filtering out data that are not reliably detected by setting a threshold for fraction of arrays (in at least one treatment group) in which the probe set is called Present by the MAS5 algorithm is a simple, easy to implement approach that works well to reduce false positives with little cost in loss of true positives. It is superior to using the average signal or RMA value because it is more likely to preserve probe sets turned on or off and it removes probe sets that show cross-hybridization. Another advantage is that filtering by fraction Present is much easier to implement, because general guidelines can be set based upon the experiment size instead of having to examine the distribution of expression values.

Filtering by fraction Present improves both parametric (Welch's t-test) and non-parametric analyses (*e.g. *SAM). For t-tests, this type of filtering rarely removes very significant probe sets (p ≤ 0.001). Although similar results in FDR improvement can be achieved filtering by average signal or RMA value, approaches based on expression level are more likely to remove genes that are being turned on or off. Permutations of data expected to produce no true positives resulted in fewer false positives than predicted by the Benjamini and Hochberg method [1], and demonstrated that probe sets called Absent produce a disproportionate fraction of false positives.

Using fold change by itself for filtering is problematic. Probe sets that are all or mostly Absent (with low signals) can generate large fold changes (due to dividing by values near zero) that are not reproducible, and represent false positives.

Smaller experiments benefit from filtering with a threshold of 0.5 or higher (a higher threshold of fraction Present); criteria can be relaxed for larger experiments although filtering is still of substantial value. Setting the threshold to 100% Present (all probe sets Present in at least one treatment group) is too stringent for experiments of any size, because it removes many of the genes being turned on or off and removes a large proportion of very significant genes with little improvement in FDR.

## Methods

### Experimental data

Three datasets were used for this analysis. The primary data set was taken from a previously reported experiment on the effects of 500 U/ml pegylated interferon alpha and 10 μg/ml ribavirin on peripheral blood monocytes (PBMC) in culture [16]("IFN data"). PBMC were isolated from different individuals and aliquots exposed to interferon, ribavirin, both or neither for 24 hours in culture. The individual samples were processed and each hybridized to a HGU133A GeneChip^® ^following standard Affymetrix protocols. The previous analysis of the IFN data determined that the ribavirin had no detectable effect on gene expression [16]; therefore, to increase the power to detect changes the arrays from the no treatment group and the ribavirin only group were combined to create a control group of 10 samples, and the interferon and interferon + ribavirin groups were combined to create an interferon-treated group of 10 samples [16].

The second dataset is from a study that compared gene expression in liver from male rats fed a diet deficient in vitamin A to a control group fed the same diet plus vitamin A ("vitamin A data") [15,21]. That study used Affymetrix RGU34A GeneChips. Data for all 14 arrays are available from Gene Expression Omnibus at NCBI, accession numbers GSM27430 through GSM27443 and GSE1600.

GeneChips^® ^for both of these experiments were processed in The Center for Medical Genomics at Indiana University School of Medicine. Affymetrix Microarray Suite 5.0 was used to generate signal values and detection calls and the data were exported for further analysis. Both of these data sets were globally scaled to a target of 1000; default parameters were used for the detection call algorithm. We also used the R package "Affy", which is part of the Bioconductor Project [23], to calculate an alternate measure of expression level using the default parameters for the robust multichip average (RMA) algorithm [17,18].

The third dataset was taken from the GEO database, GDS accession number GDS534 and reported by Spira, *et al*. [22]. This study compared bronchial epithelia from humans who were current smokers, never smoked or were former smokers. From this data we selected 20 samples from the current smokers and never smoked groups ("smoking data"). Three samples were omitted because their correlation coefficients with samples in the same group were particularly low, GSM15689 and GSM15692 (current smokers) and GSM15728 (never smoked). The current smoker samples used were GSM15684-GSM15688, GSM15690, GSM15691, GSM15693, GSM15695-GSM15702, and GSM15704-GSM15707; never smoked samples were GSM15718-GSM15727 and GSM15729-GSM15738. These samples were analyzed using MAS5 and were scaled to target intensity of 100. The GEO contained signal and detection p-value. We generated detection calls from the detection p-value using the MAS5 defaults (Present: p < 0.04, Marginal: 0.04 ≤ p < 0.06, Absent: p ≥ 0.06).

### Filtering methods

Two classes of filtering methods were evaluated. Both made decisions about retaining a probe set in the analysis based on the behavior of the probe set on the arrays in at least one treatment group. If the probe set passes the filter criteria in either group, all signal values for that probe set are retained for both groups regardless of the signal value or detection call for any individual array. Note that this retains probe sets Present in one treatment group and Absent in the other; these can be very interesting biologically. When a probe set does not meet the threshold for either group it is rejected, and signal values for all samples are removed for this probe set. The two methods were applied to data generated by MAS5 and RMA separately. For comparison, filtering was also done using the fraction called present for all arrays.

### Filtering by fraction Present

The first method used the fraction called Present in at least one treatment group to filter. For each probe set on each array, MAS5 provides a detection call, Absent (A), Present (P) or Marginal (M), which indicates whether the specific mRNA is detectable. The detection call in MAS5 is based on a non-parametric statistical test (Wilcoxon signed rank test) [5] of whether significantly more perfect matches show more hybridization signal than their corresponding mismatches. For each probe set, the number of Present and Marginal calls in each treatment group were summed using a value of 1 for Present and 0.51 for Marginal. Probe sets that met or exceeded a particular threshold of fraction Present in at least one treatment group were retained. For example using the IFN data, for a 50% fraction Present filter, probe sets were retained if the "number Present" was at least 5 in either treatment group for the 10 × 10 experiment; for 25% the number Present must be at least 2.5 in either treatment group. RMA does not provide a measure comparable to the MAS5 detection call, therefore we used the MAS5 detection call to filter the RMA data sets as well.

### Filtering by average signal

The second method used the average expression level for each treatment group for filtering and was applied to both the MAS5 and RMA datasets For each probe set, MAS5 signals and RMA values were averaged for each treatment group separately. A probe set was retained if the average expression level (MAS5 signal or RMA value) met or exceeded the selected threshold value in at least one treatment group. For example, using a filter of 100 for the MAS5 signal, probe sets are retained if the average signal for either treatment group met or exceeded 100.

### Comparison of methods

We compared the two methods when comparable numbers of probe sets were retained for analysis. To do this, for each of the selected fraction Present thresholds, we calculated the number of probe sets retained and set an average threshold for either MAS5 signal or RMA value that retained a comparable number of probe sets. For example, using the IFN data, 10,678 probe sets were retained after filtering by a fraction Present threshold of 50%; therefore we selected an average signal of 475 to retain 10,675 probe sets and an average RMA value of 5.03 to retain 10,687 probe sets for comparison.

To compare fraction Present thresholds by treatment group to fraction Present for all arrays, a threshold for the "all arrays" method was selected such that a similar number was retained by both methods.

### Differences in gene expression

To provide the baseline for comparisons in this analysis, we performed a Welch's unpaired t-test [24] on both the RMA values and the log base 2 of the MAS5 signal values to determine if there were differences in expression level between the treated and untreated groups. Since the MAS5 signal is bounded by 0 on the left and has a long heavy tail to the right, the MAS5 data were log-transformed for the analysis to produce a more nearly normal distribution (Fig. 1). RMA is already a log-based measurement. Significant differences were also identified using the Significance Analysis of Microarrays (SAM) algorithm [19]. SAM was performed using MAS5 data both unfiltered and filtered by fraction Present at selected thresholds. All SAM analyses were done using unpaired data, 500 permutations and the 10-Nearest Neighbor Imputer. The "delta table" from each SAM analysis was used for comparisons between these data sets.

Genes which had a p-value ≤ 0.05 for the difference between groups (t-test) were evaluated for whether they were turned on or off. If the sum of Present calls differed by more than 6 in the IFN data (6 of 10) and more than 4 in the vitamin A data (4 of 7) genes were called turned "on" or "off" depending on the direction of change.

Point estimates for the false discovery rate (FDR) at particular p-values were calculated by determining how many probe sets would be expected to be significant by chance (assuming a normal distribution) and dividing that by the number of probe sets that met that p-value threshold. The FDR was calculated using the number of probe sets retained after filtering. This is similar to FDR estimates from Benjamini and Hochberg [1].

(p-value) * (number of probe sets remaining after filtering)/(number significant at that p-value)

FDR values were also calculated using the q-value program [2]. P-values generated by the Welch's t-test from unfiltered and filtered MAS5 datasets were used as input to calculate FDR values.

As another estimate of the number of false positives, we carried out balanced permutations designed such that there should be no true positives. For the IFN data, arrays were randomly selected such that 5 from each actual treatment group of 10 samples were selected for one group and the remaining 10 samples (5 from each group) were used as the second treatment group. For the smoking data, 10 arrays each were randomly selected from the non-smokers and smokers groups for the first treatment group and the remainder used for the second treatment group. Thus each permuted group had equal numbers of arrays from both groups, and the comparison is expected to show no difference. Permutation were carried out 1000 times. For each probe set, totals were kept for the number of times that a probe set met a selected combination of p-value and fraction Present threshold (p-values: 0.05, 0.02, 0.01 and 0.001; fraction Present: no filter, >0%, 25% 50%, 75% and 100%). Experiment-wide totals (total number of probe sets meeting the criteria) are an average of the 1000 permutations.

### Testing effects of sample size by permutation

In addition to the analyses performed using all of the arrays, we tested the effects of number of arrays on both true and false positives. To mimic smaller experiments testing the effects of interferon, we generated smaller virtual experiments from the MAS5 data by randomly selecting samples without replacement from within each treatment group to generate sample sizes of 3, 4, 5, 6, 7, and 8 arrays per group. 1000 permutations were completed for each sample size, and the results were tallied and averaged as described above.

To examine the false positives in smaller experiments, we generated balanced permutations from the IFN data in which equal numbers of arrays were randomly selected without replacement such that equal numbers were taken from each treatment group. 1000 permutations were completed for sample sizes 4, 6 and 8 to give an estimate of how many probe sets may be called significant by chance for each sample size.

## Abbreviations used

FDR: false discovery rate

GEO: Gene Expression Omnibus (at NCBI)

MAS5: Affymetrix Microarray Suite version 5

MM: mismatch probe

PBMC: peripheral blood monocytes

PM: perfect match probe

RMA: Robust Multichip Average

SAM: Significance Analysis for Microarrays

## Authors' contributions

JNM participated in the design of the study, performed statistical analysis and drafted the manuscript. HJE conceived the study, and participated in its design, analysis and writing. Both authors read and approved the final manuscript.
